# Spatial Variation of *Acanthophlebia cruentata* (Ephemeroptera), a Mayfly Endemic to Te Ika-a-Māui—North Island of Aotearoa, New Zealand

**DOI:** 10.3390/insects13070567

**Published:** 2022-06-23

**Authors:** Steven A. Trewick, Ian M. Henderson, Stephen R. Pohe, Mary Morgan-Richards

**Affiliations:** 1 Wildlife & Ecology Group, School of Natural Science, Massey University, Private Bag 11-222, Palmerston North 4442, New Zealand; philorheithrus@gmail.com (I.M.H.); m.morgan-richards@massey.ac.nz (M.M.-R.); 2 Pohe Environmental, Whangarei 0112, New Zealand; pohe.environmental@gmail.com

**Keywords:** biogeography, genetic diversity, interspecific variation, phylogeography, range expansion

## Abstract

**Simple Summary:**

Patterns of variation within a widespread species can provide evidence of population history. Adjacent stable populations with gene flow show clinal genetic divergence forming a pattern of isolation by distance. Populations that have grown due to an increase in potential habitat such as forest cover since the last glacial maximum will have low genetic variation showing patterns of range expansion. The mayfly *Acanthophlebia cruentata* of Aotearoa, New Zealand, is widespread in streams of North Island but absent from the cooler South Island. Mayfly nymphs are restricted to streams but adults fly, facilitating gene flow among catchments. We detected higher genetic diversity at lower latitudes of this mayfly’s range compared to most of its distribution, concordant with predictions of limited forest cover in New Zealand during Pleistocene glacial periods. A signature of recent range expansion was observed in the higher latitudes. Despite initial observation suggesting mayfly size correlated with latitude, we found sex, elevation and sampling date were significant predictors of size, and some size variation is also explained by three regional groups based on haplotype distribution.

**Abstract:**

The mayfly *Acanthophlebia cruentata* of Aotearoa, New Zealand, is widespread in Te Ika-a-Māui North Island streams, but has never been collected from South Island despite land connection during the last glacial maximum. Population structure of this mayfly might reflect re-colonisation after volcanic eruptions in North Island c1800 years ago, climate cycling or conceal older, cryptic diversity. We collected population samples from 33 locations to estimate levels of population genetic diversity and to document phenotypic variation. Relatively low intraspecific haplotype divergence was recorded among mitochondrial cytb sequences from 492 individuals, but these resolved three geographic-haplotype regions (north, west, east). We detected a signature of isolation by distance at low latitudes (north) but evidence of recent population growth in the west and east. We did not detect an effect of volcanic eruptions but infer range expansion into higher latitudes from a common ancestor during the last glacial period. As judged from wing length, both sexes of adult mayflies were larger at higher elevation and we found that haplotype region was also a significant predictor of *Acanthophlebia cruentata* size. This suggests that our mitochondrial marker is concordant with nuclear genetic differences that might be explained by founder effect during range expansion.

## 1. Introduction

Aotearoa, New Zealand, hosts a rich freshwater invertebrate fauna with high levels of endemism [1]. The distribution of freshwater species provides important regional biodiversity reflective of the country’s varied environmental conditions and dynamic geological history [2,3]. Stream invertebrate composition is considered a key indicator of anthropogenic land use (e.g., [4,5,6]), because of sensitivity to changes in environmental attributes including nutrient and sediment loading and temperature. However, before human colonisation (~800 ybp), the distribution of suitable habitat and thus potential population range and density of species was also subject to changing conditions [7]. Current species ranges and their population structure are therefore expected to reflect historical and current physical and biological factors. Phylogeography can reveal the pattern and scale of population diversity across the landscape, with the potential to reveal the dominant factors influencing biogeography [8].

The New Zealand mayflies (Ephemeroptera) comprise 57 described species, all of which are found only within the archipelago and include three endemic families [9]. Of these, 40 species are recorded in North Island and 18 (representing 11 genera and three families) appear to be North Island endemics [10,11]. One species restricted to Te Ika-a-Māui North Island is *Acanthophlebia cruentata* [12], a monotypic leptophlebiid with no near relatives in the extant New Zealand fauna. The species is, on certain morphological traits, considered similar to genera including *Aprionyx* in Africa, *Hapsiphlebia* in South America, *Atalophlebia* in Australia and *Papposa* in New Caledonia [13], but a molecular analysis of a subset of global leptophlebiid taxa lacking representatives from New Caledonia was inconclusive about placement of *Acanthophlebia* [14]. The evolutionary relationships among New Zealand Leptophlebiidae and other members of the family remains unclear. Despite its obscure ancestry, distinctive combination of traits and monotypy, *A. cruentata* is abundant and occurs widely [11,13], being recorded in all North Island ecoregions of Harding and Winterbourn ([15]). Hudson [12] did not designate type material or a type locality for *A. cruentata*, but he noted it was to be found “commonly in all the streams in the vicinity of Wellington”. It is present on offshore islands including Gt Barrier, Little Barrier and Kapiti but has never been recorded in South Island. The nymphs mostly inhabit moderate speed, hard bottomed streams with abundant overhead vegetation, and upstream native forest cover is important in determining the relative abundance of nymphs [16].

The lack of near relatives of *A. cruentata* is suggestive of a specialised ecology, and the short-lived adults with vestigial mouthparts [17] generally stay close to their place of emergence [18], yet the current geographic range suggests tolerance of relatively diverse freshwater conditions. The distribution of *A. cruentata* throughout North Island required dispersal and colonisation capacity as southern North Island is geologically young [19]. However, the species has failed to invade the cooler South Island, despite recent land and freshwater connection during the late Pleistocene [19]. In contrast, the distribution and population genetic structure of other New Zealand forest animals, including flightless birds [20] and insects [21,22], and other mayflies (e.g., *Coloburiscus humeralis*) indicates this opportunity for range expansion was realised by many species.

**Figure 1:**
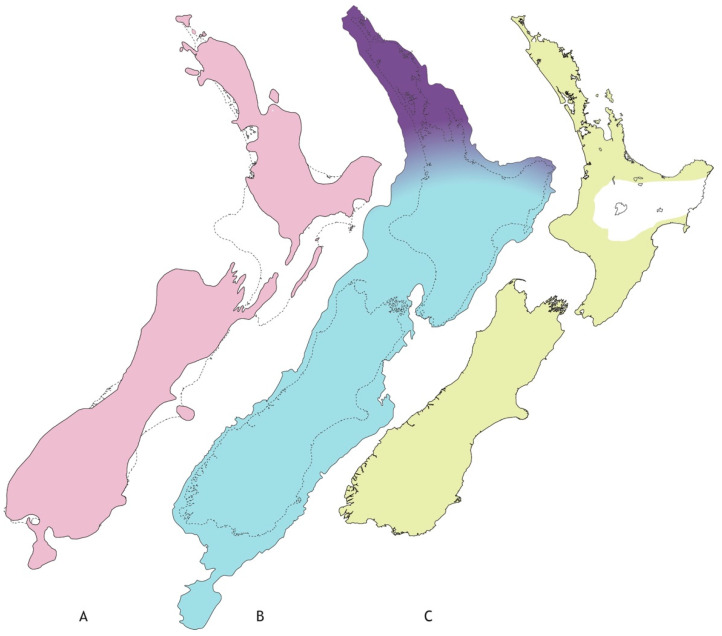
Approximate extent of landscape with freshwater habitat in New Zealand’s recent geological past. (**A**) Late Pliocene (~3Mya) with two main islands separated by marine strait(s) further north than today (shaded); (**B**) lowered sea level at Last Glacial Maximum of Pleistocene (~20Kya) following tectonic uplift of southern North Island (shaded), and limits of main forest in LGM (dark shading); (**C**) forest vegetation since LGM (shaded) with area impacted (white) by Hatepe eruption (1800ya). (Figure redrawn after: (**A**) [19], (**B**) [34], (**C**) [35].

Landscape and climatic changes are likely to influence the composition of freshwater biota and intraspecific population structure (Figure 1). In addition to the uplift of southern North Island over the last one million years, *A. cruentata* might also have been influenced by processes that altered stream physicochemistry. Indeed, reduced abundance and extirpation in streams modified by anthropogenic pasture development supports this inference [16]. In prehuman time, large-scale environmental change associated with Pleistocene climate cycling, and volcanic eruptions in the central Taupo region may have shaped *A. cruentata* range and diversity [23,24,25]. During the last glacial maximum (LGM) ending about 21 kya the cooler more arid conditions yielded an extended shrubland vegetation across much of North Island, with mixed forest being restricted primarily to lower latitude (northern) areas [26]. Southward range expansion to higher latitudes following the LGM has been revealed in some insects including the phasmid *Clitarchus hookeri* that displays the classic pattern of geographic parthenogenesis [22,27], but it is not clear how freshwater systems were affected. If the association between *Acanthophlebia* and forest streams today (cf. streams in open pasture situations) applied during the LGM (Figure 1), then we would expect a phylogeographic signature of southward population expansion. Another plausible source of population disruption is volcanism [25]. The high energy AD232 Hatepe eruption in the Taupo Volcanic Zone Taupo [28] distributed some 65 km^3^ of ash and rock over about 3000 km^2^ [29], and had a major impact on nearby biology [30]. It is expected that abrupt changes in sediment, temperature and chemistry associated with volcanic fallout would have been deleterious for freshwater life [31]. Population recovery after such events is typically characterised by lowered genetic diversity and evidence of recolonisation from elsewhere [21,32,33]. Alternatively, it is plausible that *A. cruentata* comprises cryptic diversity that would be indicated by distinct regional lineages.

Here, we examine the phylogeographic structure and adult phenotypic variation within *Acanthophlebia cruentata* across its full spatial range, to examine these possibilities. 

## 2. Materials and Methods

**Figure 2:**
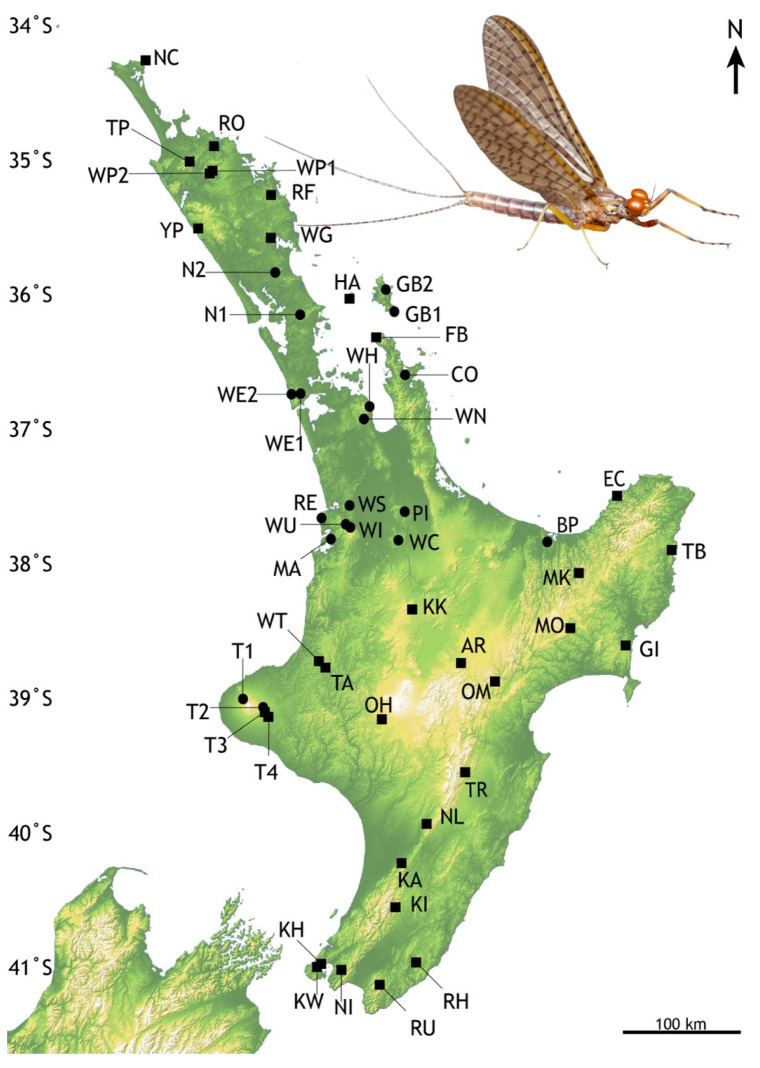
Locations in North Island, New Zealand of *Acanthophlebia cruentata* population samples. Black circles—previously reported locations [25], black squares- this study. Abbreviated codes refer to locations listed in Table 1. Degrees of latitude are given on the left; north is towards the equator. Inset: Male subimago *Acanthophlebia cruentata* from Pukenui Forest © Olly Ball and Steve Pohe Collection.

Specimens of *Acanthophlebia cruentata* were collected at small gravel streams in catchments with native vegetation, either as nymphs by hand searching stones or kick-sampling with a net, or, on emergence by UV trapping [11,36]. Specimens were preserved in ethanol for subsequent analysis. For analysis of genetic variation, we targeted locations for which published sequence data for this species were not available, particularly in the southern and northern extents of the species’ distribution. For examination of phenotypic variation of adults, we selected locations to ensure spatial coverage, but also representative altitudes across the species’ distribution (Figure 2, Table 1).

### 2.1. Genetic Variation

Ethanol preserved specimens (nymphs, subimagos or imagos) were used for whole genomic DNA extraction using a salting-out method [37]. Tissue was macerated and incubated with 10 μL of 10 mg/mL proteinase-K in 600 μL of TNES buffer (20 mm ethylenediaminetetraacetic acid, 50 mM Tris, 400 mM NaCl, 0.5% sodium dodecyl sulphate) at 55 °C for 1–4 h. 10% 5M NaCl was added and the extractions shaken vigorously for one minute before centrifuging at 16,000 g for 5 min. The supernatant was removed and precipitated with an equal volume of cold 100% ethanol. DNA was collected by spinning and washed with 70% ethanol, then air-dried and dissolved in water.

Mitochondrial DNA sequences were obtained using primers that target a portion of the cytochrome-b gene (cytb) homologous with published data for *A. cruentata* [25]. Polymerase chain reactions (PCR) used the primers CB-J-10612 and CB-N-10920 [38] in 10 or 20 μL reactions (200 μm dNTPs, 2 mm MgCl_2_, 0.5 U Taq), treated to 36 cycles of 94 °C for 30 s, 54 °C for 30 s, 72 °C for 90 s with an initial denaturation of 94 °C for 2 min and a final extension at 72 °C for 5 min. We also generated sequences for a portion of the mtDNA cytochrome oxidase subunit 1 (cox1) for a subsample of *Acanthophlebia cruentata* to provide comparative ‘DNA-barcode’ information, using PCR primers LCO1490 and HCO2198 [39].

DNA sequences were checked for ambiguities, translated and aligned in Geneious [40]. We identified distinct haplotypes including those (#1–#34) recorded by Smith et al. (2006) [25] and continued the same numbering system (#35–#52, #54–#84), for a total of 83 haplotypes. We examined the spatial distribution of haplotypes across the range of the species. Population sample locations were mapped in R [41] using the *maps* package [42] and evolutionary relationships among haplotypes were inferred with a minimum spanning network [43] implemented in PopArt [44]. During relatively slow range expansion, the populations at the leading edge might be characterized by increasingly derived sequences due to mutations accumulating on external branches [45]. However, evidence of recent recolonisation would be seen as the sharing of the same haplotypes among source and putative sink populations.

We used spatial Principal Component Analysis [46], implemented with the R package *adegenet* [47] to visualise the non-random geographic distribution of genetic variation using simple distances between DNA sequences. Spatial PCA (sPCA) utilises spatial coordinates via a connection network to explore non-random distribution of genetic variation among population samples, and uses a Monte-Carlo simulation to assess significance of structure. A useful feature of this approach is that it does not assume population groupings a priori. Based on this analysis, population samples were then grouped for analysis to identify signal consistent with hypothesised prehistoric partitioning. We used ARLEQUIN v3.5.2.2 [48] for AMOVA with population sample data grouped into three regional sets (north, west, east).

Range expansion is expected to leave a signature of increasingly divergent allelic frequencies and reduced genetic variation at the leading edge [44,49]. Southward population expansion from LGM forest refugia would result in a southward cline of reduced genetic diversity, while post-Tāupo eruption re-colonisation would result in lower diversity in central North Island with higher diversity either side. We calculated haplotype and nucleotide diversity using DnaSP v6.0 [50] and ARLEQUIN v3.5.2.2 [48] and examined population sample nucleotide diversity (π) with respect to sample location (latitude). 

Range expansion is usually associated with population growth and therefore we predicted a signature of southward demographic expansion if this mayfly species was restricted to higher latitudes in Northland during the LGM. We calculated neutrality statistics and population size changes for the entire intraspecific sample and for regional subsets (north, west, east). We looked for evidence of demographic expansion and population size constancy considering the frequency of pairwise genetic distances among haplotypes [51,52].

Genetic structure resulting from gene flow rather than range expansion would leave a signature of isolation by distance (IBD). To estimate population differentiation we calculated population sample pairwise Φ_ST_ [53] with Arlequin, and tested to see whether estimates were significantly greater than 0 using 1000 sample permutations. We compared pairwise Φ_ST_ with linear distances between sampling sites, obtained by triangulation of New Zealand Map Grid coordinates, and used a mantel test implemented in R with the package *vegan* [54] to determine whether there was a signature of IBD. 

### 2.2. Size Variation

**Figure 3:**
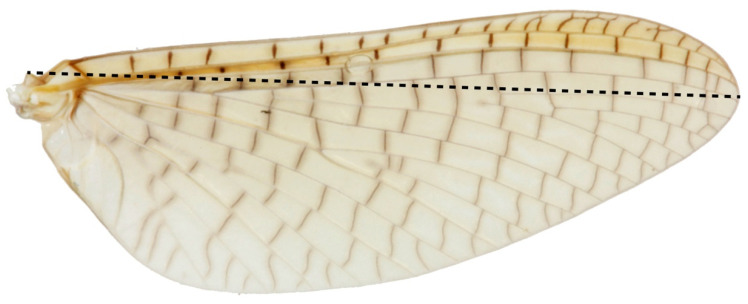
*Acanthophlebia cruentata* forewing length measurement.

We compared 1064 adult *Acanthophlebia cruentata* from 33 sites across the species range to document intraspecific phenotypic variation, using forewing length as a proxy for individual size [55]. A single forewing from each specimen was mounted under a coverslip in a syracuse dish with 80% ethanol and measured to the nearest 0.1 mm from the apex of the wing to the point of attachment at the basal plate with an eyepiece graticule through a Leica stereomicroscope (Model: MZ95) at 8–10× magnification [11]. Sampling of winged mayflies took place in one flight season between November 2013 and February 2014. All measurements were made by the same person (Figure 3). 

**Figure 4:**
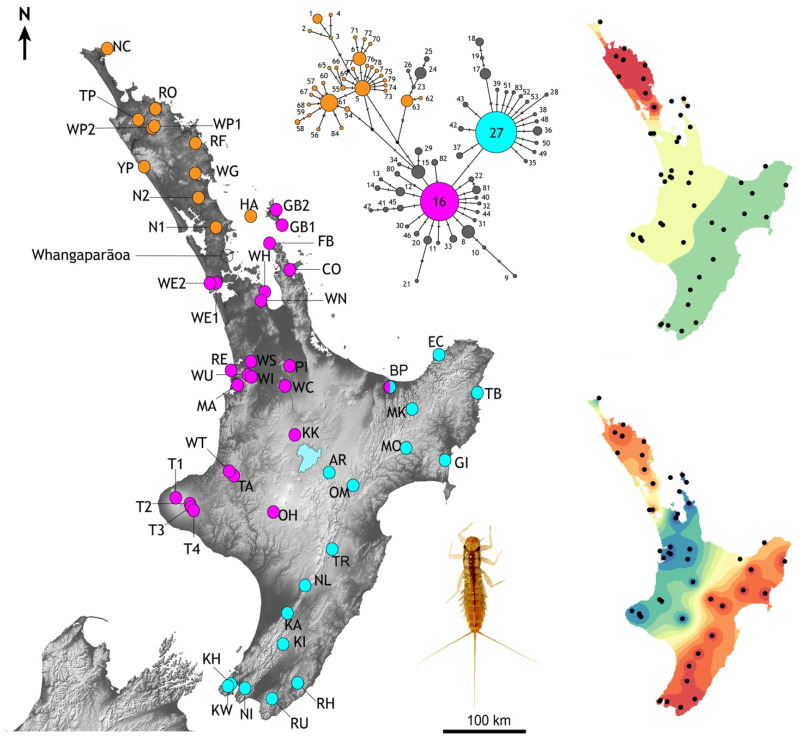
Distribution of mtDNA cytochrome-b haplotype diversity among population samples of the mayfly *Acanthophlebia cruentata* in North Island, New Zealand. Spot colours show occurrences of the two most common haplotypes (#16 magenta, and #27 turquoise), and locations in the north that lack either of the common haplotypes (orange). Scarce derivates of the two common haplotypes are grey in the network (See Supplementary Table S1). This genetic variation is also depicted by interpolations of the first two global axes of spatial PCA from these data revealing overall trends in spatial structure (**right**). Inset is late instar *A. cruentata* nymph.

Analysis used R with the package *ggplot2* [56] to plot density distributions of male and female wing lengths by sample location. Wing length was analysed with linear mixed effects models using R packages *lme4* [57] and *lmerTest* [58]. Wing length was log-transformed to remove positive skew and heteroscedasticity in residuals. Adult body size (including wing length) in mayflies is expected to be temperature dependent [59] so the covariates, elevation (100 m units), latitude (100 km units) and time of season (week after start of sampling on 22 November) were included. Preliminary plots suggested non-linear relationships of wing length with these covariates so quadratic effects were added. Sex, stage (imago, subimago) and region (based on haplotype groups, see Figure 4) were fixed factors and sampling location a random effect (R code in Supplementary Material). 

## 3. Results

### 3.1. Diversity

We obtained mtDNA cytb sequences from 308 *Acanthophlebia cruentata* individuals, from 33 population samples spanning the known range from North Cape to Wellington, North Island Aotearoa–New Zealand (Figure 1). These complemented published data representing 185 individuals from 19 other locations from part of this area [25]. The maximum population sample size for the genetic analysis was 13 sequenced individuals with a mean sample size of 9.7 and mode of 10 (Supplementary Table S1). From two sites (T4 Waingongoro, KH Khandallah) we had only a single specimen (each with a haplotype common in an adjacent location sample), these data were excluded from most analyses. This gave a data set of 492 individuals in the cytb alignment of 280 bp with 57 variable positions, comprising 83 haplotypes (49 of them new to the present study) with an overall nucleotide diversity (π) of 0.01034 (Table 2). These data were characterised by high haplotype diversity (Hd: 0.861), but low genetic divergence (mean K2P: 0.93%). New GenBank accessions ON227131–ON227179 (Supplementary Table S1).

More than 50% of all specimens from which we obtained cytb data (254/492) had one of two common haplotypes (#16 and #27), while more than three quarters of all haplotypes (85.7%) were recorded in just one or two individuals (Supplementary Table S1). One or other of the common haplotypes (#16 or #27) were observed in 80% of population samples; sixteen eastern samples with #27, and twenty-four western samples with #16 (Figure 4). Only one population sample (BP; Bay of Plenty) had both of the common haplotypes. Population samples lacking both of the common haplotypes came from Northland and Little Barrier Island (Figure 4). In the north only one sample (NC; North Cape, *n* = 8) had individuals with a haplotype very similar to the common haplotype (#80, #81 both one-step from #16; Figure 4). North of Whangaparāoa (Figure 4) the cytb haplotypes differed from one another by up to eight substitutions (2.86%), but few of these haplotypes are recorded further south. One rare exception was haplotype #45 recorded in individuals in the north (NC, *n* = 1), Coromandel Peninsular (FB, *n* = 1) and Waikato (TA, *n* = 1). 

Interpolation of the spatial distribution of the cytb genetic variation using two global sPCA axes revealed three regions with distinct genetic diversity: north, west and east (Figure 4). An imaginary line running north northeast-south southwest through central North Island (Lake Taupo) would fall between our population samples with one or other of the two common haplotypes and their derivatives (haplotype #16 in the west, haplotype #27 in the east). AMOVA indicated that more than 50% of genetic variation could be explained by the regional groups north, west and east, the remainder was explained by variation within groups (28%) and within population samples (22%), reflecting the high haplotype diversity.

### 3.2. Range Expansion

Genetic diversity as measured by the number of mtDNA haplotypes per individual within population samples (h) was generally higher in the north and northwest. For example, samples of 12 individuals from Whangarei (WG) and Waikato (TA) each had seven or eight haplotypes, while some similar sized samples from the east had a single haplotype (e.g., MO, KI; Table 2).

**Figure 5:**
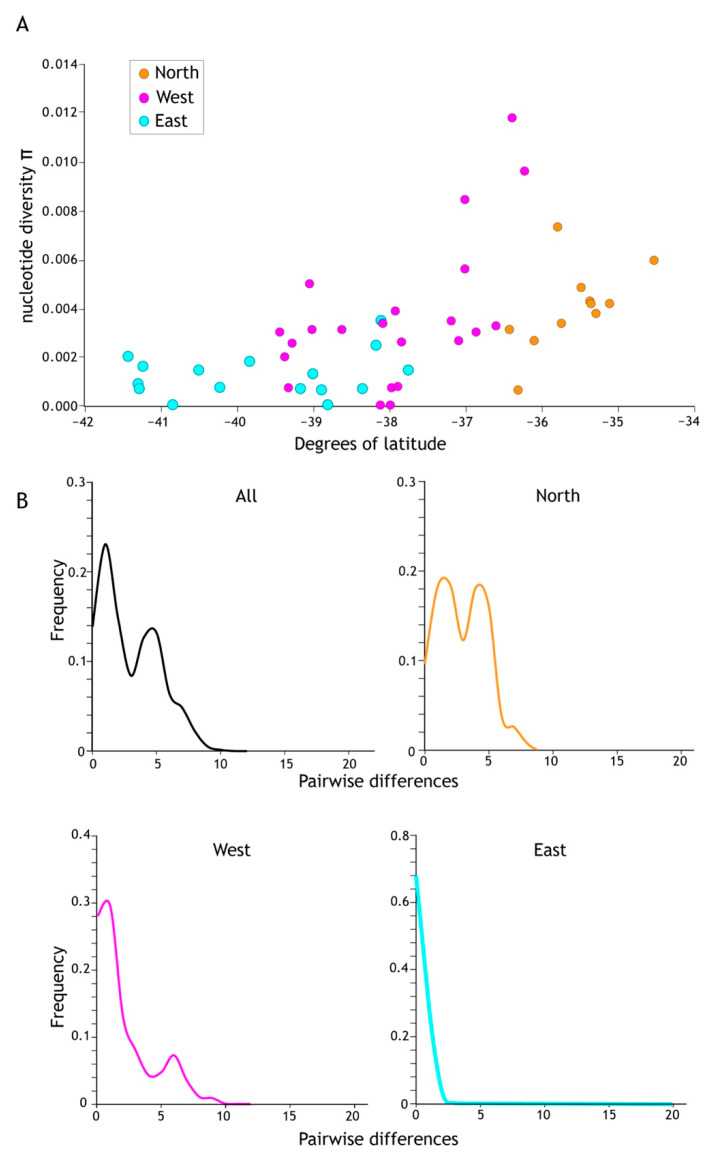
(**A**) Higher levels of genetic diversity (π) per population sample were recorded in the north of the *Acanthophlebia cruentata* distribution. Population sample nucleotide site diversity (280 bp cytb mtDNA) by latitude with north to the right and south to the left. (**B**) Mismatch distribution of cytb sequence diversity for all and regional subsets (see Figure 4).

Population sample genetic diversity (h) was lowest at the southern limit of this mayfly’s range (Table 3). Population sample nucleotide diversity (π) showed a similar pattern across North Island, New Zealand (Table 2; Figure 5A). The average π for the eleven northern population samples was 0.0105, for West 0.0073 (23 population samples) and the lowest estimate of π was in the East (0.00123; 15 population samples; Table 3) reflecting this uneven distribution of genetic variation.

Demographic analyses applied to the total cytb data set returned significant negative values for all statistics (Tajima D −1.94599 sig *p* < 0.05, Fu & Li’s D* −7.18167 sig *p* < 0.02, Fu & Lis F* −5.624 sig *p* < 0.02, Fu’s Fs −98.926), which, under the assumption of functional neutrality of the marker, is a signature of rapid recent population increase. However, when subdivided into regional population sample groups (North, West, East as in Figure 4), statistical support for expansion in the North was less compelling; Tajima’s D (−1.54881) was not significantly negative (*p* > 0.10) whereas the West (Tajima’s D −1.76828, *p* < 0.05) and East (Tajima’s D −2.35176, *p* < 0.01) were (Table 3). Viewing the data in a different way, a ragged mismatch distribution for the total data was dominated by the northern sample and indicative of a stable population whereas unimodal distributions as in the east are consistent with range expansion.

**Figure 6:**
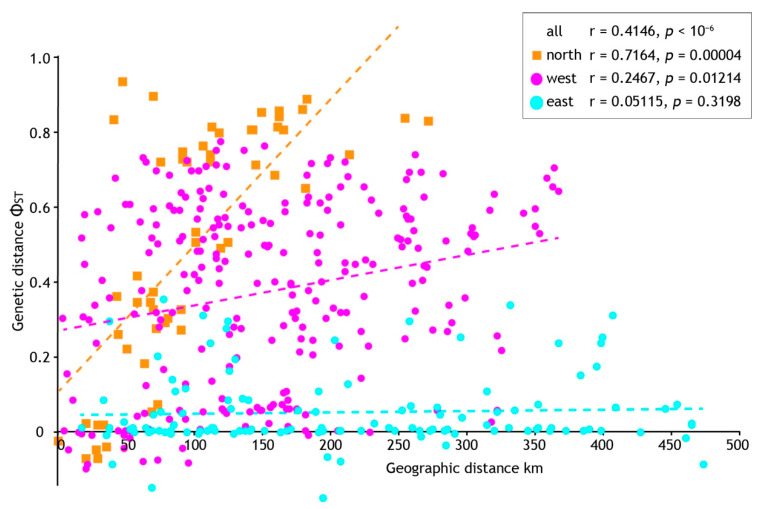
The relationship between geographic and genetic distance among population samples of *Acanthophlebia cruentata*. Results of Mantel test for all samples and geographic subsets (see Figure 4).

Analysis seeking evidence of isolation by distance (IBD) using pairwise population sample differentiation (Φ_ST_) also indicated regional-specific processes. A significant correlation between geographic and genetic distances was found when all population samples were analysed (*p* = < 10^−6^) but with a mediocre coefficient (r = 0.4146). However, we found strong statistical support (*p* = 0.00004) and a higher correlation coefficient (r = 0.7164) within the northern population samples (Figure 6). In contrast, analyses of geographic and genetic distances within the west and east population samples (see Figure 4) gave marginal (West, *p* = 0.01214, r = 0.2467) or no signal of IBD (East, *p* = 0.3196, r = 0.05115). In combination with the demographic analyses we infer longer standing population structure in the north, but recent, rapid population expansion elsewhere that is consistent with mismatch distributions.

### 3.3. DNA-Barcode Data

A subsample of 89 specimens from Taranaki (T3, WT, TA), Taupo (OH), East Cape (EC, TB), Manawatu-Wellington (KI, NI, KA, RU) and Northland (WP1, WP2) provided DNA sequence from a fragment at the 5′-end of cox1. An alignment of 585 bp contained 27 variable sites and we identified 34 distinct haplotypes (GenBank ON442277–ON442310). Among those haplotypes the average Jukes-Cantor distance was 0.00728 (0.73%) and the maximum was 0.014 (1.4%).

### 3.4. Timeframe

Mean DNA sequence divergence within *Acanthophlebia cruentata* mitochondrial haplotypes was low (K2P: cytb 0.93%, cox1 0.73%). Applying substitution rates inferred for insect intraspecific mtDNA molecular evolution of 0.0285–0.0792 substitutions per site per million years [60,61] to the median of these mtDNA distances (0.074) we can infer that the common ancestor of the diversity observed within *A. cruentata* existed approximately 38,000–50,000 years ago. Though by no means a robust time calibration, this coincides with the last glacial maximum [62] and is consistent with a southward range expansion of this species when forest habitat would have been extending and there was land connection between North and South Islands [19].

### 3.5. Wing Length—Adult Size

**Figure 7:**
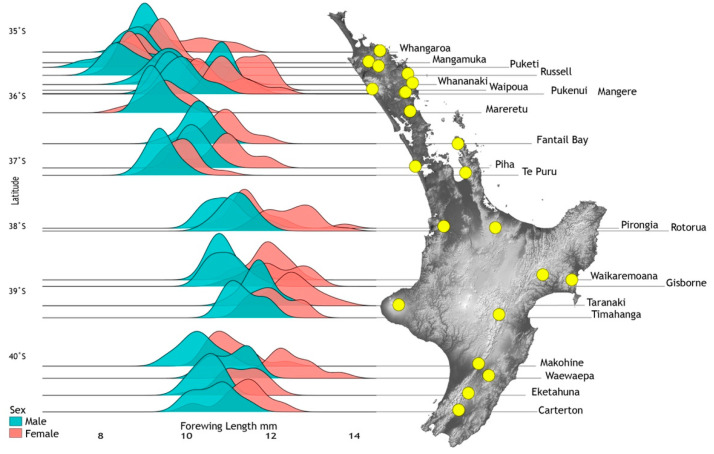
Spatial wing length variation among 21 population samples of adult *Acanthophlebia cruentata* (N = 888) at locations (yellow spots) across their latitudinal range in North Island, New Zealand. Degrees of latitude are given on the left.

Forewing lengths of 1064 individuals (imagos and subimagos) from 33 sites were measured and analysed taking into account latitude, elevation, instar and time of sampling, and lineages defined from cytb sequence data (Figure 4). Density distributions for 21 population samples with >10 individuals were graphed showing smaller size of males (Range: 7.4–12.5 mm, mean 9.96 mm, *n* = 533) compared to females (Range: 8.1–13.8 mm, mean 10.87 mm, *n* = 417) but different mean sizes across the species range (Figure 7). Data from all 33 sites were used for statistical model fitting. Attempts to fit a linear mixed effects model with interaction terms among elevation, latitude and season of sampling were abandoned as these variables are strongly confounded due to geographical constraints and the temporal pattern of sampling. In addition, the combinations of these interactions could equally be covered by the location random effect. Females were generally larger than males (0.9 mm or approximately 9%) but the response to other predictor variables was also stronger in females resulting in many significant interactions. To simplify interpretation, models that were fitted separately for males and females are reported here.

After stepwise removal of non-significant effects the final model included the random location effect (55% of variance), quadratic elevation, and haplotype region (Table 4). Male wing length is greatest at 500 m elevation where it is about 19% longer than at sea level. Locations in the northern haplotype region (Figure 4) are generally lower elevation than elsewhere in North Island but in addition to the elevation effect, northern males were 7% smaller than those in the eastern region.

**Figure 8:**
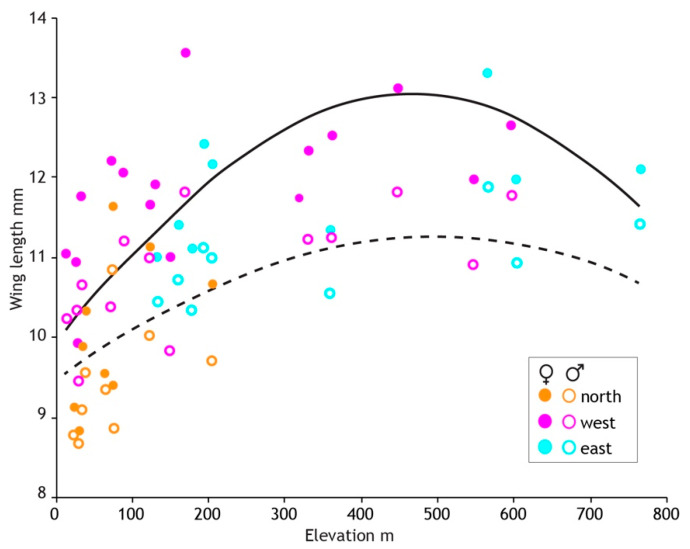
Three geographical regions defined by genetics reveal intraspecific size variation in the New Zealand mayfly *Acanthophlebia cruentata.* Average wing length and elevation (metres above sea level) at each of 33 sampling sites (N = 1064). Fitted lines (female solid, male dashed) are from the linear mixed effects models evaluated at average values of other covariates.

The results of model fitting and simplification (Table 4) were similar in females but with the addition of a weak quadratic seasonal effect. Wing length was greatest early in the season and declined with increasing slope to 5% smaller by the end of sampling 13 weeks later. The elevation and regional effects on wing length were stronger in females compared to males. Female wing length plateaued at 460 m asl, 31% longer than at sea level (Figure 8). Females in the northern region were 15% smaller than those in the eastern region. The random location effect accounted for 58% of the variance, similar to that for males. No effect of latitude was detected in either sex.

## 4. Discussion

The monotypic genus *Acanthophlebia* might have contained cryptic taxa reflective of a long history of diversification in New Zealand, but we did not find that. Instead we found low genetic divergence within *Acanthophlebia cruentata* sampled throughout its range in North Island, New Zealand; from North Cape in the Far North to Wellington in the south, Taranaki in the west and East Cape. Within the large sample of *A. cruentata* mean sequence divergence was less than 1% (cytb 0.93%, cox1 0.73%), which is at the lower end of the intraspecific range (1.2–1.4%) reported for New Zealand mayflies, stoneflies and caddisflies [63]. Other New Zealand mayflies have not been sampled at this scale (see [64]), but unpublished DNA sequences for a selection of New Zealand species sampled across their ranges revealed intraspecific variation among cox1 sequences ranging from 0.6% (*Arachnocolous phillipsi*) to 3.9% (*Zephlebia versicolor*). This contrasts with much higher sequence divergence within a number of endemic terrestrial insects in the same landscape; average mtDNA distances of ~8% in the wētā *Hemideina crassidens* [65] and *Hemiandrus pallitarsus* [66] suggest large sustained populations of these forest species.

Mayflies are habitat sensitive aquatic stream invertebrates whose populations are likely to be responsive to geophysical processes that abruptly alter local conditions [67]) and so it is not surprising that we found population structure consistent with very recent environmental changes in North Island (Figure 1) [23]. We detected isolation by distance in the northern part of the *Acanthophlebia cruentata* but a signature of population expansion in the south. Despite low divergence among the cytb sequences the haplotype diversity revealed a pattern of regionally-restricted variants delimitating three sections of the North Island. Population samples north of Whangaparāoa contained region-exclusive haplotypes and relatively high nucleotide diversity per location. In contrast, to the south, genetic diversity was lower and dominated by two common haplotypes. Here, population samples fell into east and west groups, each comprising a common haplotype and its derivatives. This pattern of haplotype distribution is typical of recent range expansion scenarios. 

Overall we found a pattern of population expansion [44,68] in central and southern North Island, contrasting with a more stable population history in Northland. The prominent east–west structure implies two parallel range expansions that likely reflect occupation of habitat in recent geological time. The directionality of range expansion within the east and west regions cannot be inferred, although the northern population samples within the western group have genetic diversity levels equivalent to the northern population samples. Within both east and west there is statistical evidence of population expansion but not isolation by distance (Figure 6). It is probable that the phylogeographic structure of *Acanthophlebia cruentata* reflects changing habitat opportunities during Pleistocene climate cycling, and we found that the level of genetic diversity indicated a common ancestor about 38,000–50,000 years ago. This suggests climate during the last (Otiran) ice age (~115,000–11,700 years ago) reduced population size of this mayfly, and the impact may date to the coldest phases associated with ice advances since ~65,000 years ago [69]. All putative genetic diversity accumulated in previous interglacials or prior to the Pleistocene has been erased, leaving this species on a relatively isolated phylogenetic lineage with shallow clade depth. As a result we find no signal for cryptic diversity or population structuring associated with land formation in southern North Island [19]. Similarly, we do not find strong evidence that volcanic eruptions at Taupo influenced local mayfly population structure (see sPCA Figure 4) despite deep ignimbrite deposits [70]. This implies a rapid reoccupation of streams around the crater from surviving populations to the east and west, although we note few unique alleles in population samples from sites near Taupo (KK-Kakaho #47; OM-Omarowa #51: Supplementary Table S1).

In New Zealand, a pattern of high intraspecific mtDNA diversity in Northland and less to the south is found in the tree wētā *Hemideina thoracica* with a concordant decline in nucleotide diversity (π) southwards [21,71]. A number of other New Zealand forest species have a genetic signature of northern refugia, suggesting a similar response to climate shifts, including the stick insect *Clitarchus hookeri* [27], and giraffe weevil *Lasicrhynchus barbicornis* [72]. Genetic signatures of population expansion were not resolved in the cicada *Kikihia cutora* but three mtDNA clades were identified with distributions that closely match the north, west, east locations of the genetic groups within *A. cruentata* [73]. Interestingly, levels of intraspecific haplotype diversity are higher in all these examples, suggesting that population reductions during periods of inhospitable conditions were more intense in *Acanthophlebia cruentata* than these other endemic insects. Given the possibility that the time of population expansion for these different species was not concordant we cannot conclude that the processes involved were the same (Cf [73]).

Intraspecific size variation in stream insects generally correlates with sex and water temperature associated with latitude and elevation, with smaller winged, small bodied adults (of each sex) at warmer (northern) latitudes and lower elevation [59,74]. This is largely the case for *Acanthophlebia cruentata,* with smaller wings observed from warmer streams, but after accounting for sex and elevation, our models found no support for an effect of latitude. Adult size, measured here as forewing length, is greater in females and they also show stronger responses to other predictors of wing length than males. Wing length was greater at higher elevation as expected from the thermal equilibria hypothesis [59], but unexpectedly declined at the highest elevation sites. Maximum wing length was observed at a similar elevation for males and females (500 m and 460 m) but there are only five sample locations above this elevation (max 775 m) so it could be simply a ‘plateau’ effect. In females there was a significant decline in wing length through the flight season which is also expected from the thermal equilibrium hypothesis as later emerging mayflies would be exposed to higher temperatures during nymphal growth. The three geographical groups (regions defined by cytb haplotypes) are strongly confounded with these proxies for temperature. The populations in the east were sampled later in the flight season and also included sites at higher elevation, especially compared to the northern region; nevertheless haplotype region was a significant predictor of *Acanthophlebia* wing length even in competition with strong proxies for temperature and after accounting for sampling location effects. This significant effect of haplotype region in our data, hinting at a genetic difference between *Acanthophlebia cruentata* in the north, west and east, might be explained by founder effect on functional genes [75]. 

## 5. Conclusions

The mayfly *Acanthophlebia cruentata* has, in the context of endemic insects of Aotearoa–New Zealand, shallow lineage diversity. Nevertheless, we are able to identify from the distribution of genetic variation a spatial pattern of late Pleistocene age. We find signal for two, parallel, range expansions within the mtDNA data and an indication that adult mayfly size is linked to distinct, though shallow, regional haplotype clusters. Changing habitat availability associated with Pleistocene climate cycling provides the parsimonious explanation for this shallow population history but it is plausible that the *Acanthophlebia* lineage itself has a shallow ancestry in Aotearoa–New Zealand. 

## Figures and Tables

**Table 1:** Sampling of *Acanthophlebia cruentata* in North Island, New Zealand ordered by latitude. Shaded entries relate to population samples reported here, and others are as given by Smith et al., (2006) [25]. N is the number of individuals sequenced from that location. Latitudes and longitudes are in decimal degrees from the New Zealand Geodetic Datum 2000 system. Region is the inferred grouping of population samples.

Site	Stream/Location	N	Catchment	Region	Latitude	Longitude
NC	Whiriwhiri	8	Great Exhibition Bay	north	−34.41618	173.02423
RO	Waiarakau, Whangaroa	10	Pekapeka Bay	north	−35.01494	173.71780
TP	Tapapa	7	Mangamuka	north	−35.190923	173.479886
WP1	Un-named	10	Waipapa	north	−35.268508	173.69025
WP2	Opaopao	13	Waipapa	north	−35.275884	173.684116
RF	Punaruku, Russell Forest	11	Whangaruru Harbour	north	−35.39779	174.31114
YP	Waipoua	12	Waipoua	north	−35.65149	173.55684
WG	Un-named, Pukenui Forest	12	Whangarei	north	−35.70250	174.26365
N2	Kaikowhiti	10	2 Manganui	north	−36.026502	174.324863
HA2	Haowhenua	6	Hauraki Gulf	north	−36.204798	175.050593
HA1	Awaroa	6	Hauraki Gulf	north	−36.226593	175.083970
N1	Waitaraire	10	1 Hoteo	north	−36.343918	174.570645
GB2	Un-named	10	6 Un-named	west	−36.15232	175.422072
GB1	Un-named	10	5 Un-named	west	−36.316537	175.53457
FB	Fantail	12	Fantail Bay	west	−36.524012	175.329540
CO	Whangapoua	9	8 Whangapoua	west	−36.790841	175.606381
WE1	Nihotupu	9	3 Nihotupu	west	−36.936229	174.558453
WE2	Marawhara	10	4 Marawhara	west	−36.940233	174.467602
WH	Whaharau	10	7 Whaharau	west	−37.030701	175.27899
WN	Mangatangi	9	10 Waikato	west	−37.124688	175.223497
WS	Whatawhata	11	10 Waipa; Waikato	west	−37.777573	175.070591
PI	Piakonui	9	11 Piakonui	west	−37.827104	175.626659
RE	Te Rekereke	10	12 Te Rekereke	west	−37.869378	174.768631
WU	Paiaka	10	9 Waitetuna	west	−37.915366	175.028061
WI	Kaniwhaniwha	10	10 Waipa; Waikato	west	−37.937943	175.074245
MA	Mangaroa	8	13 Mangaroa	west	−38.026209	174.876355
WC	Mangatautari	10	10 Waikato	west	−38.053945	175.563346
KK	Kakaho	7	Waikato	west	−38.569143	175.717612
WT	Waitara	7	Waitara	west	−38.96487	174.756806
TA	Tangarakau	12	Whanganui	west	−39.006367	174.818337
T2	Katikara	11	16 Katikara	west	−39.224687	173.966536
T3	Waipuku	9	Waitara	west	−39.288286	174.175361
T1	Patea	10	15 Patea	west	−39.324293	174.19069
T4	Waingongoro	1	Waingongoro River	west	−39.36037	174.227564
OH	Mangawhero (Ohakune)	11	Whangaehu	west	−39.400426	175.41362
EC	Whanarua	5	Un-named	east	−37.679337	177.765742
BP	Wainui	10	14 Wainui	east	−38.052137	177.06847
TB	Waihi	8	Tokomaru Bay	east	−38.105271	178.351447
MK	Manganuku	11	Opatato/Waioeka	east	−38.289889	177.385076
MO	Ruapapa	12	L. Waikaremoana	east	−38.754575	177.162035
GI	Te Arai	12	Gisborne	east	−38.82820	177.78960
AR	Te Arero	10	L. Taupo; Waikato	east	−38.955119	176.226307
OM	Omarowa	11	Mohaka	east	−39.110777	176.563837
TR	Triplex	12	Tukituki	east	−39.783281	176.26527
NL	Matanganui (No 1 Line)	11	Manawatu	east	−40.185977	175.868259
KA	Kahuterawa	10	Manawatu	east	−40.472337	175.608022
KI	Kiriwhakapapa	11	Ruamahanga	east	−40.808855	175.545166
RH	Waipunga (Rocky Hills)	9	Pahaoa	east	−41.214914	175.77028
KH	Tyers (upper), Khandallah	1	Wellington Harbour	east	−41.241352	174.787612
KW	Kaiwharawhara	12	Kaiwharawhara	east	−41.265569	174.75777
NI	Nikau	8	Wainuiomata	east	−41.27658	174.973306
RU	Ruakokoputuna	11	Ruamahanga	east	−41.403603	175.36374

**Table 2:** Summary of variation among partial mtDNA cytochrome-b haplotypes for population samples of the New Zealand mayfly *Acanthophlebia cruentata*, ordered by region (π, nucleotide diversity; h, number of haplotypes; Hd, haplotype diversity).

Site	Region	N	h	Hd	π
N1	north	10	4	0.5333	0.0031
N2	north	10	3	0.6000	0.0026
WP1	north	10	6	0.7778	0.0041
WP2	north	13	5	0.6282	0.0042
TP	north	7	3	0.6667	0.0037
YP	north	12	6	0.6818	0.0034
HA1	north	12	2	0.1667	0.0006
NC	north	8	3	0.6071	0.0059
RF	north	11	6	0.8000	0.0048
RO	north	10	5	0.6667	0.0041
WG	north	12	8	0.8485	0.0073
WE1	west	9	4	0.6944	0.0056
WE2	west	10	3	0.7333	0.0083
GB1	west	10	4	0.7111	0.0117
GB2	west	9	4	0.6944	0.0095
WH	west	10	3	0.6000	0.0026
CO	west	9	4	0.6944	0.0030
WU	west	10	2	0.2000	0.0007
WN	west	10	4	0.5333	0.0034
WC	west	9	1	0.0000	0.0000
WS	west	11	5	0.6182	0.0026
WI	west	10	1	0.0000	0.0000
PI	west	9	2	0.2222	0.0008
RE	west	10	2	0.5333	0.0038
MA	west	8	3	0.6071	0.0033
T1	west	9	2	0.5556	0.0020
T2	west	12	3	0.6212	0.0025
T3	west	10	2	0.2000	0.0007
WT	west	7	3	0.6667	0.0031
TA	west	12	7	0.8636	0.0049
OH	west	11	3	0.4727	0.0030
KK	west	7	2	0.2857	0.0031
FB	west	12	4	0.6364	0.0033
BP	east	10	3	0.6444	0.0034
EC	east	5	2	0.4000	0.0014
TR	east	12	4	0.4546	0.0018
NL	east	10	2	0.2000	0.0007
KA	east	10	3	0.3778	0.0014
KI	east	11	1	0.0000	0.0000
RH	east	9	3	0.4167	0.0016
KW	east	11	2	0.1818	0.0007
NI	east	8	2	0.2500	0.0009
OM	east	11	2	0.1818	0.0007
AR	east	10	2	0.3556	0.0013
MK	east	11	2	0.1818	0.0007
TB	east	8	3	0.6071	0.0024
RU	east	11	4	0.4909	0.0020
GI	east	12	2	0.1667	0.0006
MO	east	12	1	0.0000	0.0000
Total Data Estimates		490	83	0.8611	0.0103

**Table 3:** Summary statistics of regional variation among partial mtDNA cytochrome-b sequences from *Acanthophlebia cruentata* mayflies in New Zealand (π, nucleotide diversity; k, average number of nucleotide differences; S, number of segregating sites; h, number of haplotypes; Hd, haplotype diversity), and ‘neutrality’ statistics that can indicate population size change. Significantly negative values of Tajima’s D (bold) imply population expansion under the assumption of locus neutrality. R2 represents the relationship between singleton mutations and average nucleotide difference with a smaller value (bold) consistent with a scenario of population expansion. A larger value (bold) of the Raggedness statistic r indicates a poor fit with the assumption of constant population size. Regional groupings as shown in Figure 4 and Table 1.

	N	π	k	S	h	Hd	Tajima’s D	*p*	Ramos-Onsins & Rozas’s R2	Raggedness r
All	492	0.01034	2.8951	63	83	0.8606	**−1.9460**	**<0.05**	0.0246	0.0270
north	115	0.01045	2.9616	31	36	0.9040	−1.5488	>0.10	0.0441	0.0307
west	207	0.00731	2.0459	32	34	0.7197	**−1.7683**	**<0.05**	0.0307	0.0337
east	162	0.00126	0.0354	17	17	0.3145	**−2.3518**	**<0.01**	**0.0178**	**0.2263**

**Table 4:** Explaining intraspecific size variation of a New Zealand mayfly. Linear mixed effects model for log wing length in male (left) and female (right) *Acanthophlebia* following model simplification by stepwise removal of non-significant terms. *p*-values are from type III anova and Satterthwaite’s method for approximating degrees of freedom.

	MALE		FEMALE	
	Fixed Effects Fitted Parameters	*p*	Fixed Effects Fitted Parameters	*p*
Intercept	2.26370	<2 × 10^−16^	2.38685	<2 × 10^−16^
Week^2^			−0.00073	0.04461
Elevation	0.07004	3.047 × 10^−5^	0.11694	5.946 × 10^−7^
Elevation^2^	−0.00706	0.001713	−0.01258	2.375 × 10^−5^
Region		0.001240		0.0004293
north	−0.07063		−0.14241	
west	0.02076		0.02467	

## Data Availability

DNA sequence data are available via Genbank, accession numbers ON227131–ON227179. Wing measurement data are summarised in the text and available from the authors.

## Supplementary Materials

The following supporting information can be downloaded at: https://www.mdpi.com/article/10.3390/insects13070567/s1, Table S1: *Acanthophlebia cruentata* cytb haplotype frequencies; R code script for linear mixed effects models using R packages *lme4* and *lmerTest.*
