# Supplementary material for: Spatial Variation of Acanthophlebia cruentata (Ephemeroptera), a Mayfly Endemic to Te Ika-a-Māui—North Island of Aotearoa, New Zealand
Source: Insects. 2022 Jun 23;13(7):567. doi: 10.3390/insects13070567 (PMC9316242; doi:10.3390/insects13070567)

		Location	1	2	3	4	5	6	7	8	9	10	11	12	13	14	15	16	17	18	19	20	21	22	23	24	25	26	27	28	29	30	31	32	33	34	35	36	37	38	39	40	41	42	43	44	45	46	47	48	49	50	51	TOTAL
HAPLOTYPE	Genbank #		N1	N2	BP	GB1	GB2	T1	T2	RE	MA	WN	WH	WE1	WE2	WU	WC	WI	CO	WS	PI	OH	T3	T4	EC	TR	NL	KA	RH	NI	KH	KK	OM	AR	MK	TB	TA	KW	WT	KI	WP1	WP2	TP	RU	YP	HA	NC	FB	RF	RO	GI	WG	MO	
1	AY508417		7																																																			7
2	AY508418		1																																																			1
3	AY508445		1																																																			1
4	AY508423		1																																																			1
5	AY508421			3																																					1		2		7				5			1		19
6	AY508434			6																																						1							2			5		14
7	AY508441			1																																																		1
8	AY508430											7	6																																									13
9	AY508437											1																																										1
10	AY508446											1																																										1
11	AY508438												1																																									1
12	AY508419																		5																				1																	6
13	AY508420																		1																																					1
14																			1																														1							2
15	AY508422							4	5																														1		4															14
16	AY508447				5	3	2	5	6	6	2	1	3	1	3	9	9	10	2	7	8	8	8	1								6					4		2									7						118
17	AY508442													5	4																																							9
18	AY508443													2	3																																							5
19	AY508424													1																																								1
20	AY508435										5																																											5
21	AY508431										1																																											1
22	AY508449																			1	1																																	2
23	AY508425						1																																															1
24	AY508427					5	5																																															10
25	AY508426					1	1																																															2
26	AY508448					1																																																1
27	AY508429				4																				4	9	10	8	7	7	1		10	8	10	5		11		11				8							11		12	136
28	AY508428				1																																																	1
29	AY508444									4																																												4
30	AY508433															1																																						1
31	AY508432																			1																																		1
32	AY508440																			1																																		1
33	AY508439																			1																			3																	4
34	AY508436								1																																													1
35	ON227131	KAac3																										1																												1
36	ON227132	ECac1																							1										2		2		1																	6
37	ON227133	Nlac2																								1				1								1																		3
38	ON227134	KAac8																										1																												1
39	ON227135	NLac9																									1																													1
40	ON227136	Ohakac3																				1																																		1
41	ON227137	Ohakac6																				2																																		2
42	ON227138	RHac2																											1															1												2
43	ON227139	RHac11																											1															1												2
44	ON227140	T3ac2																					1																																	1
45	ON227141	TA52																																				1										1	1							3
46	ON227142	TA53																																				1		1																2
47	ON227143	KK117																														1																								1
48	ON227144	MK132																																	1																					1
49	ON227145	TR104																								1																														1
50	ON227146	TR106																								1																														1
51	ON227147	OM93																															1																							1
52	ON227148	TB140																																	1																					1
53	ON227149	RU5																																										1											1
54	ON227150	OP6																																								1	1													2
55	ON227151	OP2																																								1									1					2
56	ON227152	TA7																																										1												1
57	ON227153	OP5																																								1				1										2
58	ON227154	PO9																																									2													2
59	ON227155	OP8																																								1														1
60	ON227156	PO12																																									1													1
61	ON227157	OP4																																								5	8	4		1					6					24
62	ON227158	Hau1																																													1									1
63	ON227159	Hau7																																													10									10
65	ON227160	RF9																																																1						1
66	ON227161	Wg4																																																			1		1
67	ON227162	RO10																																																	1				1
68	ON227163	RO7																																																	1				1
69	ON227164	YP6																																												1									1
70	ON227165	RF1																																																1					1
71	ON227166	Wg10																																																			1		1
72	ON227167	Wg9																																																			1		1
73	ON227168	RF2																																																1					1
74	ON227169	RF7																																																1					1
75	ON227170	RO1																																																	1				1
76	ON227171	YP4																																												1									1
77	ON227172	YP1																																												1									1
78	ON227173	Wg1																																																			1		1
79	ON227174	Wg12																																																			1		1
80	ON227175	NC12																																														2							2
81	ON227176	NC7																																														5							5
82	ON227177	FB5																																															3						3
83	ON227178	Gi12																																																		1			1
84	ON227179	Wg5																																																			1		1
	N		10	10	10	10	9	9	12	10	8	10	10	9	10	10	9	10	9	11	9	11	9	1	5	12	11	10	9	8	1	7	11	10	12	7	12	12	7	11	10	13	7	11	12	11	8	12	11	10	12	12	12	492

Supplementary information: *Acanthophlebia* wing length analysis R code.

```
Winglength analysis R output
> library(lme4)
> library(lmerTest)

> males<-read.csv(file.choose())
> females<-read.csv(file.choose())
> malemodel<-lmer(logwing~alt+alt2+region+(1|loc), REML=F, data=males)
> qqnorm(residuals(malemodel))
> plot(malemodel)
> femalemodel<-lmer(logwing~week2+alt+alt2+region+(1|loc), REML=F, data=females)
> qqnorm(residuals(femalemodel))
> plot(femalemodel)
> summary(malemodel)
Linear mixed model fit by maximum likelihood . t-tests use Satterthwaite's
  method [lmerModLmerTest]
Formula: logwing ~ alt + alt2 + region + (1 | loc)
   Data: males

     AIC      BIC   logLik deviance df.resid
 -1751.3  -1721.4    882.7  -1765.3      526

Scaled residuals:
    Min      1Q  Median      3Q     Max
-3.7709 -0.6480  0.0454  0.5557  3.3822

Random effects:
 Groups   Name        Variance Std.Dev.
 loc      (Intercept) 0.002211 0.04703
 Residual             0.001817 0.04263
Number of obs: 533, groups:  loc, 32

Fixed effects:
             Estimate Std. Error        df t value Pr(>|t|)
(Intercept)  2.263698   0.029061 34.062663  77.894  < 2e-16 ***
alt          0.070037   0.015079 44.348310   4.645 3.05e-05 ***
alt2        -0.007057   0.002104 41.463782  -3.354  0.00171 **
regionnorth -0.070634   0.028472 28.323959  -2.481  0.01931 *
regionwest   0.020763   0.023530 29.242564   0.882  0.38476
---
Signif. codes:  0 ‘***’ 0.001 ‘**’ 0.01 ‘*’ 0.05 ‘.’ 0.1 ‘ ’ 1

Correlation of Fixed Effects:
            (Intr) alt    alt2   rgnnrt
alt         -0.660
alt2         0.471 -0.943
regionnorth -0.790  0.339 -0.172
regionwest  -0.704  0.173 -0.056  0.653
> anova(malemodel)
Type III Analysis of Variance Table with Satterthwaite's method
         Sum Sq  Mean Sq NumDF  DenDF F value    Pr(>F)
alt    0.039207 0.039207     1 44.348 21.5725 3.047e-05 ***
alt2   0.020440 0.020440     1 41.464 11.2464  0.001713 **
region 0.031067 0.015533     2 28.439  8.5468  0.001240 **
---
Signif. codes:  0 ‘***’ 0.001 ‘**’ 0.01 ‘*’ 0.05 ‘.’ 0.1 ‘ ’ 1
> summary(femalemodel)
Linear mixed model fit by maximum likelihood . t-tests use Satterthwaite's
  method [lmerModLmerTest]
Formula: logwing ~ week2 + alt + alt2 + region + (1 | loc)
   Data: females

     AIC      BIC   logLik deviance df.resid
 -1234.9  -1202.6    625.4  -1250.9      409

Scaled residuals:
    Min      1Q  Median      3Q     Max
-2.7397 -0.6722 -0.0537  0.6329  3.5283
```

```
Random effects:
 Groups   Name        Variance Std.Dev.
 loc      (Intercept) 0.003323 0.05764
 Residual             0.002379 0.04877
Number of obs: 417, groups:  loc, 33

Fixed effects:
              Estimate Std. Error        df t value Pr(>|t|)
(Intercept)  2.3868462  0.0493772 31.9986484  48.339  < 2e-16 ***
week2       -0.0007303  0.0003513 37.2089582  -2.079  0.04461 *
alt          0.1169416  0.0204835 50.6716291   5.709 5.95e-07 ***
alt2        -0.0125768  0.0026896 48.3721702  -4.676 2.38e-05 ***
regionnorth -0.1424129  0.0462732 30.8704207  -3.078  0.00435 **
regionwest  -0.0246753  0.0404182 31.9109987  -0.610  0.54586
---
Signif. codes:  0 ‘***’ 0.001 ‘**’ 0.01 ‘*’ 0.05 ‘.’ 0.1 ‘ ’ 1

Correlation of Fixed Effects:
            (Intr) week2  alt    alt2   rgnnrt
week2       -0.698
alt         -0.122 -0.436
alt2         0.123  0.292 -0.940
regionnorth -0.883  0.662 -0.062  0.070
regionwest  -0.850  0.711 -0.205  0.173  0.815
> anova(femalemodel)
Type III Analysis of Variance Table with Satterthwaite's method
         Sum Sq  Mean Sq NumDF  DenDF F value    Pr(>F)
week2  0.010278 0.010278     1 37.209  4.3203 0.0446099 *
alt    0.077537 0.077537     1 50.672 32.5933 5.946e-07 ***
alt2   0.052019 0.052019     1 48.372 21.8666 2.375e-05 ***
region 0.047966 0.023983     2 30.766 10.0814 0.0004293 ***
---
Signif. codes:  0 ‘***’ 0.001 ‘**’ 0.01 ‘*’ 0.05 ‘.’ 0.1 ‘ ’ 1
>
```

Plots of residuals, females top row, males bottom row.

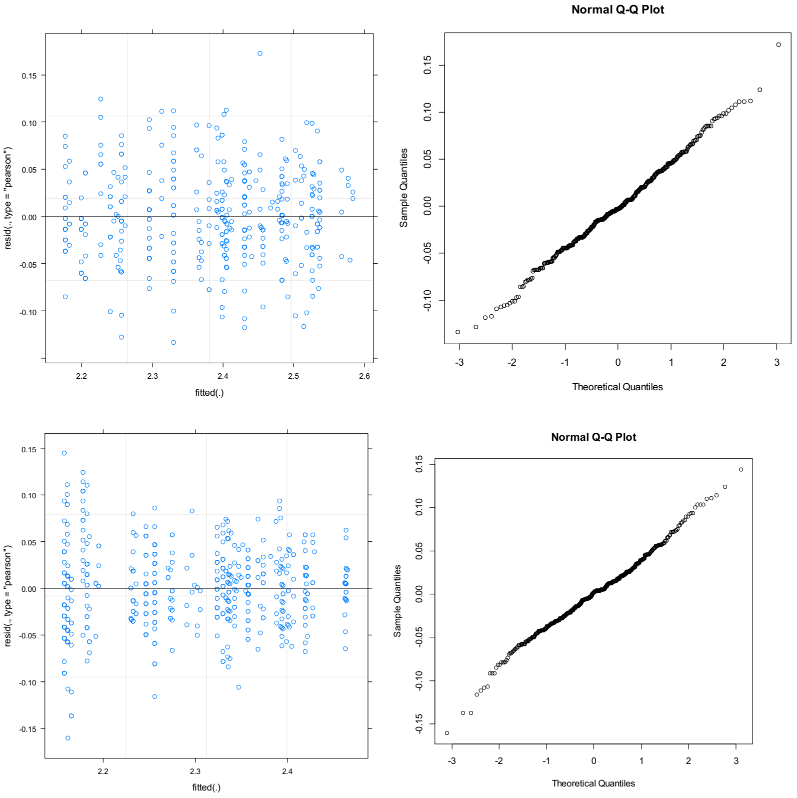

Supplement: Supplementary file 1 [file insects-13-00567-s001.zip › insects-1755102-supplementary.pdf]
